# Posologicol Table

**Published:** 1879-07

**Authors:** 


Posological Table—Including all the officinal and the most frequently employed unofficinal preparations. By Chas. Rice, Chemist Department of Public Charities and Correction, N. Y., etc. Revised and approved by members of the Medical Boards of Bellevue and Charity Hospitals. Wm. Wood & Co., N. Y., 1879.

This is a neat little book of ninety-six pages, and is replete with information on the doses of remedies, mode of application, form of preparation of drugs, and a comparison of the different systems of weights and measures. A very convenient little book to have handy for ready reference.

